# Norwegian “dugnad” as a rhetorical device in public health communication during the COVID-19 pandemic. A qualitative study from immigrant’s perspectives

**DOI:** 10.1186/s13690-024-01237-0

**Published:** 2024-01-19

**Authors:** Raquel Herrero-Arias, Irina Vladimirovna Halbostad, Esperanza Diaz

**Affiliations:** 1 Department of Health Promotion and Development, University of Bergen, Bergen, Norway; 2 Migration healthcare service, Namsos municipality, Norway; 3 Pandemic Center, Department of Global Public Health and Primary Care, University of Bergen, Bergen, Norway

**Keywords:** COVID-19, Pandemic, Immigrants, Norway, Health communication

## Abstract

**Background:**

During the COVID-19 pandemic, the Norwegian government appealed to the term “national dugnad” in the communication of containment measures as a call for collective action to fight the spread of infection. “Dugnad” is traditionally associated with solidarity, social responsibility, and a communal spirit in the form of volunteer work carried out by a local community. Although the word “dugnad” is difficult to translate to other languages, it was used as a rhetorical device by the government to communicate health-related information during the pandemic. This study aims to explore how immigrants understood and related to the term “dugnad” as used in the context of the COVID-19 pandemic in Norway.

**Methods:**

We conducted 55 semi-structured interviews in 2020 with immigrants from Poland (10), Syria (15), Somalia (10), Sri Lanka (10), and Chile (10). Interviews were conducted in participants’ mother-tongues. We used systematic text condensation following Malterud’s four steps to analyze the data.

**Results:**

The results are organized into three themes corresponding to: (1) meaning making of the term “dugnad”; (2) attitudes towards the term “dugnad”; and (3) reactions to the use of “dugnad” in a public health context. Overall, participants were familiar with the term “dugnad” and positively associated it with volunteering, unity, and a sense of community. However, we found a variety of reactions towards using this term in a public health context, ranging from agreement to disagreement and irritation.

**Conclusion:**

Health communication during pandemics is crucial for maximizing compliance and gaining control of disease spread. In multicultural societies, governments and authorities should be aware of the linguistic and cultural barriers to public health communication if they are to effectively reach the entire population. The use of culturally specific concepts in this context, specially as rhetorical devices, may hinder effective health communication and increase health inequalities.

**Supplementary Information:**

The online version contains supplementary material available at 10.1186/s13690-024-01237-0.

**Table:** 

**Text box 1. Contribution to the literature**
• Public health communication during a health crisis should be sensitive to cultural and linguistic diversity.
• In Norway, the government used the culturally and linguistically specific concept of “dugnad” to call for solidarity and collective engagement during the COVID-19 pandemic.
• Despite positive attitudes towards the term “dugnad” and the COVID-19 measures among a diverse group of immigrants in Norway, the results provide evidence that the government’s use of culturally specific terms as rhetorical devices in public health communication may hinder effective health communication and increase health inequalities.

## Background

With over 600 million confirmed cases, including over 7 million deaths, the COVID-19 pandemic has been the worst global health crisis of the century [1]. In Norway, the numbers of deaths and hospital admissions due to COVID-19, relative to population size, were lower than in many other countries, and the government managed to control the situation rather quickly [2, 3]. In March 2020, the Norwegian government introduced the strictest peacetime measures ever, which included the closure of day care centers, schools, and higher education institutions, and restrictive regulations on domestic travel. There were also recommendations for social behavior, such as physical distancing, hand washing, quarantine, and avoiding public transport [4].

Public health became an international priority. Under great pressure to manage this health crisis, leaders worldwide used rhetorical strategies in their communication of the measures aimed at maximizing compliance. Although political rhetoric varied between countries, war metaphors and expressions to encourage communal cooperation were frequently used in public health communication [5, 6]. In Norway, the government appealed to people’s collective responsibility and sense of community by using the traditional expression “dugnad”, which is central to Norwegian culture and reflects the cultural values of togetherness and solidarity [7–9]. *Dugnad* comes from the Old Norse *dugnaðr*, which means “help” or “support.” Lorentzen and Dugstad [10] analysed the history and use of this term and concluded that it has four elements: it involves (a) unpaid work, (b) where people meet face-to-face, (c) in tasks that have a defined start and end point, and (d) that are followed by a social gathering. Dugnad refers, therefore, to voluntary, but strongly encouraged and expected, unpaid work for the local community. It is a concept that inculcates community engagement action as well as social control [11]. Dugnad as a social activity is often performed in kindergartens, neighborhoods, and children’s sports venues by parents, residents, and children themselves, and it usually ends with a communal meal. This concept was chosen as Norway’s national word in 2004 and has been central to the Norwegian moral repertoire of the socially responsible citizen [11].

From March 2020, the Norwegian government actively used the word “dugnad” and the phrase “a call to a national dugnad” in their public health communication calling for citizens’ collective responsibility and togetherness as individuals and as a nation to cope with COVID-19 [7]. “National dugnad” was first used in press conferences by the prime minister. Eventually, this term was actively used in formal documentation sent from the Directorate of Health to local council leaders, which promoted the extended use of this term in the pandemic context and its acceptance by the public [9]. Most people from the majority population accepted the use of “dugnad” in public health communication and related to the sense of solidarity and community that it appeals to [9, 11]. It is worth noting that the activities usually associated with the term “dugnad” changed when this term was applied in public health communication during the pandemic. In contrast with the traditional social activities associated with this term, during the pandemic “dugnad” referred to engaging in activities that required social distancing [11].

However, the word “dugnad” may not be well-known outside of the Norwegian cultural context [11]. Norway is a multicultural country where 18% of the population is of immigrant origin, with over 200 nationalities with varying languages and cultural and social traditions [12]. Therefore, it could be expected that some groups of the population, like immigrants, found it challenging to embrace the use of a culturally specific concept in a public health emergency context. Moreover, Norwegian language proficiency is of great importance for the promotion of good health among immigrants [13]. This became more visible during the pandemic, as low Norwegian proficiency could be a barrier to access information on public health measures, which were communicated in Norwegian at the beginning of the outbreak [14, 15]. Language barriers, together with socio-cultural and economic factors, have been identified as important aspects of the overrepresentation of immigrants in the rates of infection and hospitalization in Norway and internationally [16–20]. The aim of this study is to explore how immigrants understood and related to the term “dugnad” as it was used in the context of public health communication during the COVID-19 pandemic.

## Materials and methods

### Design, sample, and recruitment

This study is part of Inncovid.Norge, a research project initiated by the University of Bergen in April 2020 with the aim of enhancing knowledge of immigrants’ access to information, understanding of risk management, and ways of dealing with illness during the first phase of the COVID-19 pandemic in Norway [14, 15, 21]. The Inncovid.Norge research team consisted of researchers with immigrant background whose mother-tongues were Spanish, Somali, Arabic, Tamil, and Polish.

Fifty-five participants (26 men and 29 women), originally from Somalia (10), Syria (15), Sri Lanka (10), Chile (10) and Poland (10) and living in Norway were recruited for this study. This sample size was considered appropriate because it provided thick descriptions to achieve richness in the thematic analysis [22]. We included in our study one participant who was born in Norway because he/she used his/her mother-tongue, Tamil, in her everyday to a much larger extent than the Norwegian language. As shown in Table 1, participants were diverse in terms of age, education, and length of residence in Norway.

**Table 1:** Sociodemographic characteristics of the participants

	Gender	Age category	Country of origin/ parents’ origin	Education	Period of stay in Norway (years)
1	F	41–50	Poland	university	16
2	F	41–50	Poland	high school	9
3	M	31–40	Poland	high school	10
4	M	31–40	Poland	secondary	15
5	F	51–60	Poland	university	5
6	F	21–30	Poland	secondary	4
7	F	41–50	Poland	university	3
8	M	51–60	Poland	university	11
9	F	61–70	Poland	university	39
10	F	31–40	Poland	university	8
11	F	51–60	Chile	secondary	16
12	M	51–60	Chile	secondary	32
13	M	31–40	Chile	secondary	11
14	F	61–70	Chile	basic	25
15	F	41–50	Chile	secondary	32
16	M	21–30	Chile	secondary	9
17	F	61–70	Chile	secondary	40
18	M	31–40	Chile	master’s student	1
19	M	31–40	Chile	university	10
20	F	61–70	Chile	secondary	32
21	F	61–70	Syria	high school	5
22	M	41–50	Syria	secondary	3
23	F	61–70	Syria	high school	4
24	M	41–50	Syria	primary	3
25	F	31–40	Syria	high school	6
26	F	41–50	Syria	secondary	4
27	F	21–30	Syria	high school	3
28	F	31–40	Syria	university	9
29	M	41–50	Syria	no school	2
30	M	51–60	Syria	secondary	7
31	F	41–50	Syria	high school	5
32	M	31–40	Syria	primary	1
33	M	41–50	Syria	high school	5
34	F	61–70	Syria	high school	5
35	M	41–50	Syria	secondary	5
36	F	21–30	Sri Lanka	university	Born in Norway
37	M	31–40	Sri Lanka	university	16
38	M	51–60	Sri Lanka	high school	39
39	F	31–40	Sri Lanka	university (bachelor)	15
40	M	51–60	Sri Lanka	high school	31
41	M	51–60	Sri Lanka	high school	30
42	F	51–60	Sri Lanka	high school	35
43	M	41–50	Sri Lanka	high school	10
44	M	41–50	Sri Lanka	university (bachelor)	11
45	F	81–90	Sri Lanka	no school	8
46	F	41–50	Somalia	primary school	22
47	F	31–40	Somalia	primary school	12
48	F	41–50	Somalia	university	31
49	M	41–50	Somalia	high school	13
50	F	21–30	Somalia	high school	10
51	M	41–50	Somalia	secondary	17
52	M	51–60	Somalia	high school	32
53	M	51–60	Somalia	bachelor	30
54	M	41–50	Somalia	university (bachelor)	6
55	F	51–60	Somalia	high school	25

To recruit participants, researchers used their personal networks and approached key members of immigrant communities to ask them to spread the word among immigrants who might be interested in participating. They advertised the study on Facebook groups used by immigrants in Norway and employed snowballing, that is, asking participants to recommend others who fit the recruitment criteria. Participants who wanted to participate in the study contacted the researchers by phone.

Semi-structured interviews were conducted in April and May 2020 by telephone because of restrictions on face-to-face meetings. Interviews lasted from 30 to 45 minutes and were conducted in the native language of the participants. The interview guide included 14 questions (see appendix) on topics including sources of information about the pandemic, risk perception, authorities’ measures and recommendations to prevent rapid growth in infection, and psychological impact of the pandemic. The interview guide was developed in Norwegian by researchers from the Inncovid.Norge team, who later translated the questions into their mother tongues (i.e., Somali, Arabic, Tamil, Spanish, and Polish). Assistance from professional interpreters was offered for the successful management of this task. All topics included in the interview guide were addressed in all interviews, however in varying degrees in terms of depth. The final question was: “*Norwegians often talk about ‘dugnad’ in connection with the measures taken to prevent the spread of COVID-19. What do you think when you hear the word ‘dugnad’?”* This question assumed that the participant was familiar with and understood the original meaning of “dugnad” and was able to relate it to the handling of COVID-19. When this was not the case, each interviewer would explain the meaning of “dugnad” and ask the participant about his/her thoughts regarding the application of this concept to the pandemic context.

### Analysis

The interviews were audio-recorded, transcribed verbatim, and thereafter translated into Norwegian by professional interpreters and bilingual project assistants with proficiency in Norwegian and participants’ mother-tongues. As we were interested in exploring the understandings and attitudes participants had towards the word “dugnad” and its use in the context of COVID-19 restrictions, we coded the parts of the transcripts that were relevant to our research question for this paper. Analysis was conducted by systematic text condensation following Malterud’s four steps: familiarization with the data; identifying units of meaning that were relevant to our aim and encoding them; decontextualizing and condensing similarly coded units of meaning; synthesizing and recontextualizing the content within the coded groups by developing descriptions of the participants’ experiences and perspectives [23].

Following an inductive approach, codes and preliminary themes emerged from the data. Our analysis also followed a semantic approach that focused on the meaning explicitly communicated by participants [24]. In this process, all authors contributed with insights to the review and formulation of the themes.

### Ethics

Inncovid.Norge received ethical approval from the Norwegian Regional Ethics Committee (project number 132585). Participants were provided with written and verbal information in their native language about the study and gave consent to participate in the study before being interviewed.

## Results

The results are organized into three themes corresponding to: (1) meaning making of the term “dugnad”, (2) attitudes towards the term dugnad, and (3) reactions to the use of “dugnad” in a public health context.

### Meaning making of the term “dugnad”

Most participants were familiar with the culturally specific concept of “dugnad” and defined it in line with its original meaning as identified by Lorentzen and Dugstad [10]. In this way, the participants associated the term “dugnad” with collective and voluntary work that supports community-building. Particularly, participants highlighted volunteerism as a central aspect of this concept, which contributes to solidarity and social responsibility. *“I really like it (the concept of “dugnad”) because it’s not compulsory in any way or established by any organizations. It’s fine that it comes from the needs of ordinary people. It shows that people are sensitive to each other” (Participant 9)*.

A sense of community, solidarity, shared responsibility, and cooperation, which were seen as pillars of civil society, were other positive words used to define “dugnad”. *“It (dugnad) is something that is absolutely necessary. After all, we cannot claim our rights until we have done our duties. (…) A good society can’t be run without ‘dugnad”. “The thing is that, as far as Tamils is concerned, we have dugnad. Moreover, the situation that has come up has also contributed to “dugnad”; even if we did not have democracy, we have this thing about working together. Even if the politics deceive us, we have this value of cooperation with us. You have to do it, or it will not be good neither for us nor for the nation. I think that the best is to do what we can do for each other and there is also a joy in it” (Participant 38)*.

Participants from Somalia and Sri Lanka were familiar with the Norwegian term “dugnad” and could also identify an equivalent word in their mother tongues. However, among Syrians, there were more participants who had never heard this word before. *“We have our own word for this in Somali. (when I heard the word “dugnad”), I think about volunteering and cooperation and joint efforts for the society. Doing things together that everybody can contribute to” (Participant 48).*

When defining “dugnad”, participants referred mostly to values and the traditional activities associated with the term like group work done by neighbors (e.g., tidying and cleaning a local area followed by a communal meal).

*“When I hear ‘dugnad’, it means I have to go out, for example, in the autumn and rake up leaves with my neighbors” (Participant 8).*

*“Now, I remember, I’ve heard this (dugnad) when you live in a block of flats and all the neighbors go out and clean the outside areas… and then they cook something to eat” (Participant 15).*

### Attitudes towards the term “dugnad”

Across the interviews, we found positive attitudes towards the word “dugnad” and the values and actions it refers to. *“It’s a nice word, a positive word” (Participant 11).*

Participants especially emphasized the sense of togetherness and the positive contributions that engaging in “dugnad” would bring to the local community and wider society. *“It (“dugnad”) is very important; it helps the society, and it is positive” (Participant 24)*. *“And thanks to this dugnad, as you can observe in housing cooperatives, we all get a nice place to live, one that is clean” (Participant 4*).

When asked about this term, most participants who were familiar with it recalled pleasant memories from occasions in which they had participated in “dugnads”: *“I know that word. I’ve heard of it before. Well, I’ve observed it and I like it. I’d say I like it a lot…” (Participant 7)*.

*“Actually, this word of dugnad, I heard it for the first time when I lived in an apartment where we had this twice a year, that you worked together in a group, and when I was involved in doing these things together, I realized people came together and worked voluntarily.” (Participant 44).*

### Reactions to the use of “dugnad” in a public health context

When asked about the use of the word “dugnad” by the government in their communications regarding COVID-19 measures, most participants expressed that the application of this culturally specific concept in a public health context made sense. In this regard, they referred to the importance of bringing the society together to control the spread of the virus. *“A person should cooperate with the society as a whole, to reduce the spread of the disease, and spread information about protection from the virus and try to control himself and people around him” (Participant 25).*

“*In this context (the pandemic), I think it (“dugnad”) is about everyone’s responsibility. “Dugnad” means that everyone must work together to stop or prevent the spread of the virus. Everyone, from the young to the elderly, and everyone must participate together, get involved as a joint effort” (Participant 42).*

Participants pointed out that it made sense to use this concept because COVID-19 was considered a *“national problem that affects us all and we all have to contribute” (Participant 46)*. Those who agreed with the use of “dugnad” by the government and public health authorities stressed that a successful handling of the pandemic required collective action and those who would not participate in this would ruin the effort. *“I think it makes sense to use this word now because it is something collective, something that must be done for the common good. And if it is not done collectively, it won’t work” (Participant 13).*

However, in their discussions on the government’s use of “dugnad”, participants highlighted that the activities traditionally associated with the word “dugnad” changed during the pandemic. This difference could lead some to be sceptical about the application of the word in the pandemic context. *“Well, yes, I understand that the issue is that we should do some ‘dugnad’ in terms of, well… we make a joint effort to maintain social distancing and make sure that people who might be at risk don’t get ill…, well, as I said, I like the concept and I actually think it’s a good thing, but paradoxically, as I said, I always connected the idea of ‘dugnad’ with what we have to do on these dates or a bit before May the 17th (the Norwegian national day), like tidying and washing things in the community where I live, in the building, and that is not happening (during the pandemic)” (Participant 19).*

Across the interviews, we found participants who disagreed with the use of “dugnad” in government communications about restrictive measures to deal with COVID-19. These participants thought that complying with the measures was an action that would primarily benefit the individual by protecting him/herself from infection. *“… but I think this word ‘dugnad’ is used wrongly here because you do your duty… above all you’re helping yourself, you wash your hands, you protect yourself, you help yourself and your family, so what kind of ‘dugnad’ are you doing?” (Participant 12)*.

Disagreements over the use of “dugnad” in the pandemic context were also found among participants who questioned the voluntariness and sense of community regarding compliance with restrictive measures. *“How can we work together without getting together and how can doing things this way help us to get rid of the disease?” (Participant 32).* Moreover, we found that the use of “dugnad” in connection with public health measures on a national level could lead to a feeling of irritation. This was the case of participant 37 who highlighted the local scope of the term “dugnad”, according to which people who live close to each other engage in activities and build relationships with each other. Based on this, he questioned its applicability in the pandemic context. “*When I hear that word (dugnad), it irritates me. That word annoys me because, well, your home, if your home is a flat, then you have to do a “dugnad” where the flat is. That’s where you do a “dugnad”. If you’re going to have a “dugnad” everywhere, then it’s a word that annoys me. When it’s used in that way, it just causes even more irritation, to be honest”.*

## Discussion

“Dugnad” is a culturally specific Norwegian term that has no equivalent in many other languages [11]. In connection with COVID-19, the word was used as a rhetorical device by the Norwegian government and health authorities to appeal to the population’s sense of collective effort in a public health crisis [9]. In this article, we explore immigrants’ understandings and attitudes towards the application of this concept in a public health communication context. We were interested in their views regarding the use of this word to communicate governmental COVID-19 measures and whether it contributed to embracing the message that everyone should engage in a “national dugnad” to stop the pandemic.

Language acquisition is a complex, dynamic process influenced by level of education, native language, health problems, motivation, learning opportunities, and personal characteristics. All of these factors can affect the level of integration, understanding of Norwegian culture, and acquisition of important health competencies [25, 26]. In our study, most participants were familiar with the term “dugnad” in its original sense. This was particularly the case among participants from Sri Lanka and Somalia, who had an equivalent word in their mother tongues and resided in Norway for an average of 20 year. This group of informants also identified the moral repertoire of the socially responsible citizen in which “dugnad” is embedded [11], in their cultures of origin despite differences regarding political and welfare state contexts. Among Syrians, we found more participants who had never heard of “dugnad” before. This group was diverse in terms of gender and age and had lived in Norway for 2 to 7 years. However, based on our findings, we cannot report a clear association between longer length of stay in Norway and familiarity with the concept of “dugnad”, since Chilean and Polish participants who had never heard this word before had lived in Norway for anywhere from 1 to 25 years. Regarding attitudes towards the general term “dugnad”, we found positive attitudes from all participants because, according to them, this concept refers to positive values of solidarity, unity, and a sense of community. Many shared positive experiences of engaging into voluntary activities with their neigbors or other parents at the school.

Rhetorical devices are used in government communications to evoke emotional responses in the public. During the pandemic, use of rhetorical devices to appeal to shared social norms could be seen as a successful communication strategy, as it promotes community engagement [27, 28]. In Norway, the use of “dugnad” was aimed at evoking norms of social responsibility and cohesion. Studies have confirmed that the majority population embraced this narrative and their understanding of the pandemic as a collective problem motivated their compliance with COVID-19 measures [8, 9, 11]. We also found that several participants were pleased with the government’s use of the term “dugnad” and used this framing to help them understand the virus as a global threat that required collective action. As Moss and Sandbakken found in their interviews with people from the majority population [9], this group of participants in our study pointed out that people who would not join this collective action would ruin the effort. However, contrary to previous research [8, 9, 11], we also found opposition to the government’s use of “dugnad”, despite all participants both agreeing with the country’s COVID-19 measures and having positive attitudes towards the original concept of “dugnad”. Although it is challenging to understand the reasons for this opposition in terms of associations with “being a migrant” or single factor, life trajectories and individual experiences of the migrants, particularly interpersonal relationships built in Norway, decisively shape their attitudes and trust in the government’s management of the pandemic [15]. These could also explain why we found a variety of reactions towards the use of “dugnad” in a public health context, ranging from agreement to irritation across the data, irrespective of age, gender, length of stay in Norway, and educational backgrounds.

To better understand this, it is important to look at the activities that “dugnad” in the COVID-19 context referred to, such as social distancing, good hygiene, and isolation. As our participants mentioned, these activities differed from those typically associated with “dugnad” [9, 11]. This variance could lead to confusion over and disagreement with the use of the word during the pandemic. Moreover, some participants understood that complying with the measures was a duty or an individual action that elicited benefits to oneself, which contradicted the original meaning of “dugnad” as a voluntary action performed for the common good. These disagreements were not found in studies that conducted interviews with individuals from the majority population although the context for the application of “dugnad” was the same [8, 9]. Here, we must ask what role migration might play in participants’ responses. During the pandemic, immigrant groups experienced increased discrimination [29, 30], something that we also found in our data as we reported in another analysis [15]. Paradoxically, while the government used the term “dugnad” to foster feelings of togetherness, public discourses blamed immigrants for the rising infection rates and pointed at their cultures and lack of integration for not engaging in “dugnad” [31, 32]. This could be a key factor influencing how immigrants handled communication from the government. Research has found that groups that experience discrimination are more likely to be sceptical about public health information [28]. Furthermore, the appeal to social norms in public health communication can reinforce the exclusion and marginalisation of groups who may not identify with those norms [27, 33]. In our study, this could partially explain why some participants were critical of the idea that everyone was in the same boat and should engage in “dugnad”. Using “dugnad”, which is a culturally specific term, can, therefore, lead to greater polarization of society where vulnerable groups have a hightened sense of being outsiders [34, 35].

Our study has several weaknesses. Although our participants were selected from five language groups and were heterogeneous in terms of family situation, immigration background, length of stay in Norway, fluency in Norwegian, and cultural and socio-economic status, they are presented as if they belong to a common group of “immigrants” in this project. We acknowledge that this group is not homogeneous and that our population is not representative of all immigrants in Norway. When participants reported that they had not heard the word “dugnad” before, each interviewer explained the meaning of this word to them. We did not prepare a standardized definition for participants, which could have affected their responses; however, all interviewers were familiar with the term “dugnad” and had jointly discussed its use in public health communication when they developed the interview guide.

## Conclusion and implications

The use of a culturally specific concept to appeal to shared values and norms and persuade people to comply with public health measures can hinder effective health communication and increase inequalities. Furthermore, the extent to which culturally specific communication may reinforce immigrants’ exclusion should be explored in more detail. When communicating public health measures, it is important that politicians and other authorities are aware of and acknowledge diversity within a country’s population, particularly, diversity of social and cultural norms and forms of communication. This is imperative during a health crisis like a pandemic, as effective public health communication is key to reduce infection rates and control disease spread.

### Electronic supplementary material

Below is the link to the electronic supplementary material.

Supplementary Material 1

## Data Availability

We include the interview guide as an Additional File. The dataset is not publicly available because the participants did not consent to the data being made available to other researchers. However, additional qualitative data analysis from our dataset are being published elsewhere.

## References

[1] WHO. WHO Coronavirus (COVID-19). Dashboard 2023 [Available from: https://covid19.who.int/?gclid=Cj0KCQjw9_mDBhCGARIsAN3PaFNVuA3kABELXjY66cXUIVcTNNBkPrX57w1OtZL1GYBomSnvTRflQTcaAoxkEALw_wcB.

[2] Tvedten HM, Holtermann MK, Vikum EF. Folkehelsen etter covid-19. Del 1: Pandemiens viktigste helsekonsekvenser [Public health after COVID-19. Part 1: The most important health consequences of the pandemic]. 2022.

[3] Christensen T, Laegreid P (2020). Balancing Governance Capacity and Legitimacy: how the Norwegian government handled the COVID-19 Crisis as a high performer. Public Adm Rev.

[4] Norwegian Government. The Coronavirus Situation. 2020. https://www.regjeringen.no/en/topics/koronavirus-covid-19/id2692388/ [accessed October 10, 2023].

[5] Semino E (2021). Not soldiers but fire-fighters - metaphors and Covid-19. Health Commun.

[6] Montiel CJ, Uyheng J, Paz ED (2021). The Language of Pandemic leaderships: Mapping Political Rhetoric during the COVID-19 outbreak. Polit Psychol.

[7] Arora S, Debesay J, Eslen-Ziya H. Persuasive narrative during the COVID-19 pandemic: Norwegian Prime Minister Erna Solberg’s posts on Facebook. Humanit Soc Sci Commun. 2022;9(35).

[8] Sandbakken EM, Moss SM. Now we are all in the same Boat. At the same time, we are not. Meaning-making and coping under COVID-19 lockdown in Norway. Hum Arenas, 2021, 1–25.

[9] Moss SM, Sandbakken EM. Everybody needs to do their part, so we can get this under control. Reactions to the Norwegian government Meta-narratives on COVID-19 measures. Polit Psychol. 2021. 10.1111/pops.12727 PMC8014765 33821063

[10] Lorentzen H, Dugstad L (2011). *Den Norske Dugnaden: historie, Kultur Og Fellesskap* [The Norwegian dugnad: history, culture, and Community].

[11] Nilsen ACE, Ann; Skarpenes O (2020). Coping with COVID-19. Dugnad: a case of the moral premise of the Norwegian welfare state. Int J Sociol Soc Policy.

[12] SSB. Fakta om innvandring (Facts about immigration) 2023 [Available from: https://www.ssb.no/en/innvandring-og-innvandrere/faktaside/innvandring.

[13] Kjollesdal MKR, Gerwing J, Indseth T (2023). Health risks among long-term immigrants in Norway with poor Norwegian language proficiency. Scand J Public Health.

[14] Czapka E, Ortiz-Barreda G, Herrero-Arias R, Haj-Youne J, Møen KA, Hasha W et al. Who is telling the truth? Migrants’ experiences with COVID-19 related information in Norway– a qualitative study. Scan J Public Health. 2022. 10.1177/14034948221135237 PMC9666409 36377047

[15] Herrero-Arias R, Czapka E, GabyOrtiz-Barreda, Diaz E. The evolvement of trust in response to the COVID-19 pandemic among migrants in Norway. Int J Equity Health. 2022;21(154). 10.1186/s12939-022-01747-9 PMC9632581 36329455

[16] Diaz E, Nørredam M, Aradhya S et al. Situational brief: migration and COVID-19 in Scandinavian countries. *Lancet Migration*; 2020.

[17] Rostila M, Cederstrom A, Wallace M, Aradhya S, Ahrne M, Juarez SP (2023). Inequalities in COVID-19 severe morbidity and mortality by country of birth in Sweden. Nat Commun.

[18] Chilunga FP, Appelman B, Vugt Mv, Kalverda K, Smeele P, Es Jv et al. Differences in incidence, nature of symptoms, and duration of long COVID among hospitalised migrant and non-migrant patients in the Netherlands: a retrospective cohort study. Lancet Reg Health - Europe. 2023. 10.1016/j.lanepe.2023.100630 PMC10079482 37261215

[19] Norredam M, Hayward S, Deal A, Agyemang C, Hargreaves S. Understanding and addressing long-COVID among migrants and ethnic minorities in Europe. Lancet Reg Health Europe. 2022;19. 10.1016/j.lanepe.2022.100427 PMC9241826 35789882

[20] Kjollesdal MKR, Juarez SP, Aradhya S, Indseth T. Understanding the excess COVID-19 burden among immigrants in Norway. J Pub Health. 2022. 10.1093/pubmed/fdac033 PMC8992298 35285905

[21] Madar AA, Benavente P, Czapka E, Herrero-Arias R, Haj-Younes J, Hasha W et al. COVID-19: access to information, level of trust and adherence to health advice among migrants in Norway. A cross sectional study. Arch Pub Health. 2022;80(15). 10.1186/s13690-021-00764-4 PMC8725426 34983639

[22] O’Reilly M, Parker N (2013). Unsatisfactory saturation’: a critical exploration of the notion of saturated sample sizes in qualitative research. Qual Res.

[23] Malterud K (2012). Systematic text condensation: a strategy for qualitative analysis. Scand J Public Health.

[24] Braun V, Clarke V (2006). Using thematic analysis in psychology. Qual Res Psychol.

[25] Einarsen KJ. Språket - første skritt mot integrering [Language - the first step towards integration]. 2013 [Available from: https://www.ssb.no/utdanning/artikler-og-publikasjoner/spraaket-forste-skritt-mot-integrering].

[26] HOD H-oo. Strategi for å øke helsekompetansen i befolkningen: 2019–2023 (Strategy to icrease health literacy in the population: 2019–2023). 2019.

[27] Hyland-Wood B, Gardner J, Leask J, Ecker UKH. Toward effective government communication strategies in the era of COVID-19. Humanit soc sci Commun, 2021, 8.1.

[28] Bavel JJV, Baicker K, Boggio PS (2020). Using social and behavioural science to support COVID-19 pandemic response. Nat Hum Behav.

[29] Spiritus-Beerden E, Verelst A, Devlieger I, Langer Primdahl N, Botelho Guedes F, Chiarenza A, De Maesschalck S, Durbeej N, Garrido R, Gaspar de Matos M (2021). Mental Health of refugees and migrants during the COVID-19 pandemic: the role of experienced discrimination and daily stressors. Int J Environ Res Public Health.

[30] Benavente P, Ronda E, Diaz E (2023). Occupation-related factors affecting the health of migrants working during the COVID-19 pandemic–a qualitative study in Norway. Int J Equity Health.

[31] Gross L. Not a major or complicated Task: activating Dugnad under COVID-19 and the imagination of Equality in the Norwegian Welfare State. Anthropologica, 2023, 651.

[32] Diaz E, Mamelund SE, Eid J, Aasen HS, Kaarbøe OM, Brokstad RJC (2021). Learning from the COVID-19 pandemic among migrants: an innovative, system-level, interdisciplinary approach is needed to improve public health. Scand J Public Health.

[33] Lupton D (2015). The pedagogy of disgust: the ethical, moral and political implications of using disgust in public health campaigns. Crit Public Health.

[34] Gullestad M (2002). Invisible fences: egalitarianism, nationalism and racism. J R Anthropol Inst.

[35] Ure KH. Den høye tilliten gir oss superkrefter. En kritisk analyse av metaforer og andre stilistiske trekk i den norske helsepolitiske diskursen om koronaepidemien [The high level of trust gives us superpowers. A critical analysis of metaphors and other stylistic features of the Norwegian health policy discourse on the COVID-19 epidemic]. [Master’s thesis]. Bergen, Norway: University of Bergen; 2022.

