# Supplementary material for: Norwegian “dugnad” as a rhetorical device in public health communication during the COVID-19 pandemic. A qualitative study from immigrant’s perspectives
Source: Arch Public Health. 2024 Jan 19;82:11. doi: 10.1186/s13690-024-01237-0 (PMC10797926; doi:10.1186/s13690-024-01237-0)
Supplement: Supplementary file 1 — Supplementary Material 1 [file 13690_2024_1237_MOESM1_ESM.docx]

**Interview Guide- InnCovid.Norge Study**

1. Have you received information about the coronavirus and the pandemic?

2. Where can you find information about the coronavirus?

3. How do you perceive risk

a) for own health

b) for the health of your loved ones

c) for the health in general for everyone living in Norway?

4. What do you know about the measures and recommendations implemented by the authorities/national measures?

5. Do you think that the measures and recommendations that have been implemented will improve the situation:

a) for your own health?

b) for the health of your loved ones?

c) for the health in general for everyone living in Norway?

6. To what extent do you and people you know follow the measures that have been implemented, for example good hygiene and limited social contact?

7. If you follow the recommendations, how do you do it?

8. The measures will be implemented for a long time. What can help you follow them over time?

9. If you or someone you know finds it very difficult to follow the recommendations:

a) what makes it difficult?

b) what would make it easier?

10. If you or someone in your family has symptoms of covid-19

a) what would you do?

b) would you report it to the authorities, so they know about it? Why/why not?

11. Is there anything regarding this situation (both the illness, the measures, the media, etc.) that stresses you? If so, would you like to say something about it?

12. Is there anything in this situation that makes you remember things you have experienced in the past? If so, can you say something about it?

13. Norwegians often talk about "dugnad" in connection with the measures taken to prevent the spread of covid-19. What do you think of when you hear the word "dugnad"?
